# Making beds in early modern England: sleep, matter and environmental change^*^

**DOI:** 10.1093/hisres/htae001

**Published:** 2024-03-19

**Authors:** Holly Fletcher

**Affiliations:** University of Manchester, United Kingdom

## Abstract

Bringing environmental history, the history of medicine and the history of poverty into conversation with material culture studies, this article argues that sleep management in early modern England involved environmental practices in which bodies and matter were interwoven. Using records relating to the Worshipful Company of Upholders in London as a starting point, the article uncovers for the first time the range of animal and plant matter upon which early modern people slept. In so doing it transforms our view of the sleeping conditions of the early modern poor and demonstrates the significance of place-specific, material knowledge for health care practices.

On 1 August 1693 the London warehouse of a leather dresser named Mr. Woodhouse was searched by officials from the Worshipful Company of Upholders.^1^ ‘Upholders’ practised the craft of upholstery and were referred to variously as upholders, upholsters and upholsterers throughout the early modern period.^2^ The warehouse in Horsleydown, Southwark, was found to contain 867 ‘sea beds’ and pillows filled with goat hair, which had been removed from the animals’ skin using caustic lime. The use of goat hair was a breach of statutes enacted in 1495 and 1551, which forbade the sale of bedding stuffed with any material other than dry-pulled feathers or clean flocks of wool or cloth.^3^ The beds were thus confiscated and transported across the river Thames to Great St. Helens, where they were claimed by Sir William Gore, an alderman in the city, who would later become lord mayor of London. According to the company records, Gore was ‘pretending [the beds] were made for the use of their Ma[jes]ties Fleet’. Indeed, in December of the previous year, Gore had written to the navy requesting payment for around 900 hammocks filled with hair, stating that since they had been specially made for the navy he could not ‘dispose of them any other way’.^4^ The navy were seemingly reluctant to take the ‘sea beds’, clearly referring to a form of stuffed hammock, due to their suspect filling. Nevertheless, Gore’s explanation for the confiscated beds led to him being granted a replevin from the sheriffs of London ‘to take those beds out of the Companys possession’, a decision with which the company vehemently disagreed. Officials ordered the company’s beadle to ‘immediately go and with the assistance of some porters guard the place where the beds [were]’ to prevent Gore from retrieving them.

This was an exceptional occurrence for the company. Two weeks later a committee was formed to manage the affair and decide what to do about the beds seized from Gore.^5^ It took time before any decisions were made and a year later another committee was formed ‘to sell and dispose of Sir William Gore’s beds’.^6^ Moreover, it was decided that six of the beds seized from Gore would be publicly ‘burnt before the Royall Exchange’, in a symbolic demonstration of his wrongful use of bedding materials. Later in the year more beds were ordered to be burnt with ‘the quantity of the filling of six beds [to] be burnt by St George’s Church on Southwarke, the quantity of four beds at the May Pole in East Smithfield, and six beds in Shadwell near Hallumb House’, while the deputy master and wardens of the company bore witness.^7^

With this act of destruction, the company of upholders marked Gore’s extreme violation of the statutes in a very public way, drawing on a long tradition of ritualized public destruction among London’s livery companies.^8^ The defacement of objects was justified in terms of upholding the collective reputation of a particular trade that would be threatened by the sale of poor-quality wares. In the process, consumers were said to be protected from unwholesome or inferior goods. Yet the staging of ritual destruction and its accompanying public humiliation was arguably as important as the eradication of the objects themselves, acting to underpin the power of the guild, and maintain its social hierarchies, through a system of enforcement and coercion.^9^ The Gore case does not fit the typical template for such ritual shaming, since he was neither the creator of the beds nor, as an alderman, in a subordinate position to the guild’s governors. Together with the extent of his transgression, this latter fact may have even encouraged the scale of the public burnings, which defied the instructions of the sheriffs of London, who had sought to return the beds to Gore, thereby intervening in the company’s affairs. What this incident makes clear, however, is just how seriously the Company of Upholders was willing to take the enforcement of laws aimed at regulating the use of proper bedding materials.

These statutes were meant to prevent the deceit of upholsterers who added cheap, low-quality materials to their beds without their customer’s knowledge, thus increasing their profits as customers paid for beds they thought were filled with flocks and feathers. They also acted to control the sale of beds and bedding within the city of London, securing the company’s monopoly on such items. Yet these sources reveal concerns that were not purely regulatory or economic. Their restrictions were framed in terms of ensuring the health of those who used such beds, and thus these legal records are imbued with ideas about the body that we might expect to find in quite different kinds of sources. Bedding made with improper stuffing was stated to be ‘contagious for Man’s Body to lie on’.^10^ Since beds were intended primarily as sites for sleeping, and as the body was believed to be particularly vulnerable to disease and other potential dangers during sleep, these statutes thus present an understanding that sleeping upon forbidden materials could have serious implications for one’s health.^11^

As Sasha Handley has shown, in early modern England restful sleep was understood to be essential for ensuring a long and healthy life, as well as for maintaining a healthy household and civil society. Practices of sleep management ‘governed the rhythms of daily life’, including efforts to secure healthy sleep through the management of sleeping environments. It was thought that bedchambers should be kept clean, cool, airy and comfortable, while the bed itself and the textiles upon it ‘represented the frontline in the battle to secure a peaceful night’s rest’.^12^ These items, which naturally came into contact with the sleeping body, had the capacity to disturb or encourage rest, as well as to spread or prevent disease and contagion, depending on their material qualities. Beds and bedding, sleep and health were thus deeply intertwined, and concerns about sickness and contagion in relation to bedding materials were certainly not confined to the upholders’ company. Handley has examined the significance of beds and their textiles within ‘the practical and emotional economy of early modern households’, showing how the provision and maintenance of beds and bedding was shaped by concerns about health and cleanliness, as well as material comfort and emotional meaning.^13^

Early modern beds consisted firstly of a bedframe, almost invariably made of wood, the base of which was typically held together by a system of interwoven cords or ropes that were knotted at the end, and which could be tightened to give the sleeper more support. Cheaper bedsteads would have had a simple sacking base, and those who could not afford a bedstead would have placed their mattresses directly on the floor. For those with a bedstead, mattresses or ‘beds’ would be layered on top of the bed cords. These were soft stuffed pads made from two rectangular pieces of ticking material, known as a bed tick, and stuffed with some form of soft filling. It was fairly typical for individuals to sleep on more than one mattress or bed per bedstead, especially in wealthier households, and each mattress had a slightly different purpose. Coarser and firmer mattresses of straw or chaff, for example, would be placed beneath softer beds of wool or feathers, thus ensuring sleepers had both comfort and support. Accompanying the bedstead and bed would be a bolster to support the head or body, typically containing the same stuffing material as one of the bed’s mattresses. There could also be a pillow to place beneath the head. On top of the mattress were laid bedsheets of various grades of linen or cotton, including holland, calico and dimity, which were then topped by a blanket, ‘coverlet’ or quilt.^14^

All of these items were expensive household goods that were highly prized and would often be passed down in personal bequests, indicating their monetary and sentimental value. Inventory-based studies of early modern beds have shown the immense cost of beds and linens in this period. Lorna Weatherill, for instance, noted that such items could represent up to half the value of all household possessions among non-elite households.^15^ Mattresses, bolsters and pillows were among the most expensive components of a bed. When Lancashire farmer Richard Latham bought a feather bed and bolster in 1724, for example, he paid two pounds and three shillings, the household’s highest financial outlay that year.^16^ Buying such items new from an upholsterer was a significant investment, therefore, and many beds and bedding items were inherited or bought second-hand. Brokers and slop sellers regularly sold second-hand beds and bedding in early modern London. The satirist Thomas Brown, for instance, noted in 1702 that ‘the bedding to be sold at the Ditchside near Fleetbridge smells of the bawdy house and brandy’.^17^ As Sarah Pennell has shown, the circulation of second-hand bedding was practised widely and across the social spectrum, making early modern beds increasingly accessible to a wider range of consumers. Craig Muldrew’s sample of labourer’s inventories shows that the proportion of the labouring poor who owned items such as feather beds and bed hangings steadily increased between 1550 and 1700, reaching around 40 per cent in each case.^18^ Also considering non-elite bedding, Pennell has demonstrated that ‘middling’ people spent considerable time and money maintaining beds and bedding in the seventeenth and eighteenth centuries, while Laura Gowing has used legal records to uncover the emotional and social resonances of ‘ordinary beds’.^19^ While these studies depart from previous scholarship, which typically focused on state beds and elite interiors, the material sleeping conditions of the poor remain largely neglected.^20^

The records relating to the Company of Upholders transform our understanding of the sleeping arrangements of the poor by bringing to light the numerous materials contained in beds, both new and second-hand, which were sold by upholsterers and other salespeople to those who could not afford more costly alternatives such as flocks or feathers. These sources show that cheaper materials were not merely used by upholsterers to deceive their customers, but also to cater specifically to the needs of the poor. They provide unique insights, therefore, into the participation of poorer people in the growing consumer culture of early modern London, addressing a significant gap in existing historiography in which the material lives of the poor have often been overlooked.^21^ Given the greater number of elite material objects that have survived in museum collections, research into the material culture of poverty typically relies on creative readings of documentary sources. John Styles, for instance, has explored the kinds of objects poor people encountered when renting cheap, furnished accommodation in London by examining theft records from the Old Bailey.^22^ More recently, Joseph Harley has analysed pauper inventories from the eighteenth century, showing how regional factors and life cycles of poverty affected patterns of consumption among a broad cross section of the poor.^23^

This article builds on such scholarship by using the records of the Company of Upholders to reveal the role of the poor, as both consumers and salespeople, in driving the production and sale of bedding items filled with cheap but comfortable bed stuff.^24^ These sources include statutes and petitions concerning prohibited materials, as well as the company’s court minute books, which detail fines issued to those who continued to use them. From these records, this article uncovers for the first time the range of materials upon which poor people in early modern England slept, showing the choice of these materials to be dictated not merely by affordability, but also comfort and health. The first section of the article outlines these sources, giving an overview of the bedding materials discussed and the reasons for forbidding them.

As well as the material culture of poverty, this article intervenes in both environmental history and the history of medicine and the body, areas of study that rarely come into conversation.^25^ By exploring the material and environmental knowledge involved in collecting and preparing bed stuff, the article builds on neo-materialist histories centred on the principle of embodied codependence between people and their material environments.^26^ This principle shaped early modern people’s conception of themselves in relation to their physical surroundings, through an awareness of the entangled materialities of human and non-human agents.^27^ Moreover, since bodies and matter were known to be deeply entwined, objects and environments were understood to have significant consequences for a person’s health. This understanding informed contemporary health care advice, in turn influencing the experience, and creation, of particular objects and environments.^28^ Examining the intersections between early modern material culture, environment and health care, this article reveals the material codependencies that shaped efforts to secure healthy sleep during this period.

Following an overview of the Company of Upholders’s sources, the remaining sections focus on three categories of bed stuffing – feathers, animal hair and plant matter – which are analysed in relation to contemporary medical and botanical knowledge, agricultural practices and animal husbandry. The article thus examines the multi-species interactions involved in sleeping well, as human health and comfort during sleep depended on engagement with a range of animal and plant species, including their cultivation and maintenance, as well as natural cycles of growth and decay. The use of particular bed fillings, which were understood to impact the quality and healthfulness of a person’s sleep, is further shown to rely on an awareness of localized ecologies, demonstrating the significance of place-specific, material knowledge for health care practices. Integral to sleep management in early modern England, therefore, were environmental practices in which bodies and matter were interwoven.

*

Efforts were first made to regulate the stuffing of beds in 1474, when members of the Company of Upholders presented the mayor of London with a petition reporting that numerous people were making and selling items, including ‘Federbeddes, pylowes, matrasses, Quysshens, Quyltes and such oth’e’, which belonged to the craft of upholsterers.^29^ Since only the exterior of such bedding was visible, one ‘knoweth not the stuff within’, meaning it could be stuffed with false materials in place of appropriate fillings such as flocks or feathers. This deception, they stated, contributed to ‘the grete hurt’ of the people, as well as the ‘rebuke of the said craft’. The upholders were clearly concerned that the sale of unwholesome wares would impact on the trade’s reputation, and it seems likely that the company was more concerned with upholding their monopoly on the sale of beds and bedding than preventing the ill health of their customers. The politics of knowledge clearly entered into their complaint, as petitioners worried about the impact that those working outside the apparatus of the company could have on its operations. It was not until 1750 that an act was passed requiring all those practicing upholstery in the city of London to become members of the company, and thus there would have been numerous makers involved in the production and sale of beds and bedding who were beyond the company’s immediate control.^30^

With regard to improper bed stuffings, the petitioners specifically mentioned ‘ffetherbeddes and bolsters stuffed with feders and flokkes, pelewes of down stuffed with thistill downe and cattes tailles, materas stuffed with here and flokkes and sold for flokkes, Materasse of netis [cow] here and hers [horse] her’, all of which were deemed unacceptable.^31^ The petitioners requested that the mayor grant them the authority to search ‘all suche wares’, and to confiscate those that were made of such improper materials. This petition was granted and regulations against false materials were written into law in 1495. The materials listed in the statute were similar to those of the petition, yet the statute expanded upon their problematic nature. In this case, the health implications of improper bedding were emphasized as they were stated to be ‘contagious for Man’s Body to lie on’.^32^ While control over the sale of bedding items may have been the company’s chief concern, the statute’s discussion of the healthfulness of bedding materials nevertheless reveals a contemporary framework in which beds, sleep and health were closely intertwined.

The statute continued by outlawing the sale of ‘Quilts, Mattresses, and Cushions, stuffed with Horse-hair, Fenn-down, Neats-hair, Deers-hair and Goats-hair, which is wrought in Lime-fats, and by the Heat of Man’s Body the Savour and Taste is so abominable and contagious, that many of the King’s Subjects [have] thereby been destroyed’. While the ‘rebuke and slander’ of upholsterers remained a concern, this act, issued by parliament rather than upholders, centred around health dangers posed by unsuitable bedding materials. It drew on broader understandings within early modern medical advice that connected the composition of sleep materials with health care. The bedding materials deemed most healthy were those that moderated airflow and body temperature. Linen, for example, was frequently used in early modern bedding as the qualities of coolness and absorbency enabled linen sheets to moderate body heat and wick away night-time excretions, in turn supporting digestion, regulating the body’s fluids, and preserving the tension of the muscles, joints and nerves.^33^ References to health in this act, and subsequent regulations, need to be understood within this wider context of concerns surrounding the sleeping body and its immediate material environment.

The 1495 act was specifically concerned with beds produced and sold outside London, stating that while the company had some ‘power and authority’ to search bedding in the city, they had no such power beyond its confines. Thus, it was stated that no bedding should be sold ‘in Fairs, ne in Markets within this [the king’s] said Realm’ unless it was stuffed with ‘dry pulled Feathers’ or ‘clean Down’ only. Anyone who was selling ‘unlawful’ wares, rather than possessing them ‘for their own proper Use in their Houses’, would forfeit them. Crucially, therefore, people were free to use alternative materials in beds for their own household use, but upholsterers and other tradespeople were not permitted to sell beds made from such materials. It also becomes clear from this act that the issue at stake was not merely the use of improper materials, but also the mixing of materials. Simply combining feathers and flocks, both of which were deemed to be suitable bed stuffs, could be ‘contagious’.

A second act, issued in 1551, expanded on the list of unsuitable materials for filling beds, which included ‘scalded Feathers, Fen-down, Thistle-down, Sand, Lime, Gravel, Hair or any other unlawful or corrupt Stuff ‘.^34^ This act also enforced the payment of a moiety, or tax, on all forfeitures to the king and his successors. The statute contained some familiar materials from earlier regulations, including fen-down and thistledown, which were the fluffy seeds of cattail plants and thistles, respectively. New additions included sand and gravel, which were related to concerns about the deceitful practices of upholsterers since feather beds were valued based on their weight.^35^ The purposeful inclusion of sand and gravel in beds sold by upholsterers was intended to increase a bed’s weight, in turn increasing its value.

The next update to the regulation of bedding materials was issued via the company’s Royal Charter of Incorporation from 1626. Again it was stated that beds were being sold containing ‘divers corrupt stuffes and wares of sundry sorts not wholesome for mans body’.^36^ Such ‘stuffes’ included

scalded feathers and drie pulled feathers together, flockes and feathers together, stinking and dustie feathers mingled with soape ashes, quills, pigeon feathers, greene sick feathers, fenne downe, thistledown, rubbish, hatmakers wooll, strick haire, horsehair, neateshair, and goates haire.

The list of unsuitable stuffing materials was thus lengthened further, and this extract evidences the role of the senses for evaluating such materials, highlighting those feathers that smelled bad and had a dusty texture and green colour. The issuing of the Royal Charter itself originated from the need to provide company officers with the rights to more extensive searches of bedding being sold in London and beyond. The charter granted the company the power to create laws to govern all those practising the art of upholstery in London, as well as ‘any other Citty or place within our said Realme of England’.^37^ They could also institute searches of goods being sold within a seven-mile radius of London and in all ‘faires or marketts’ in England.^38^ While the company was better able to control the sale of beds and bedding within London itself, in theory its jurisdiction reached much further.^39^

When the company’s bylaws were issued in 1679, however, they applied only to those within seven miles of London. This may have reflected the difficulty of enforcing such rules across a wider geographical area. Within the city, all those who sold beds but who did not have a ‘sale shop’ had to report their location and wares to the company for inspection. Again, this suggests measures were taken to control the sale of bedding by those outside the guild’s infrastructure, since this rule targeted street sellers and other casual traders, rather than formally trained upholsterers with commercial premises. It was by the former salespeople that the company suggested ‘the trade be abused’.^40^

The bylaws also restated the list of prohibited materials. These included a mix of flocks and feathers, and ‘corrupt or stinking feathers’. All feathers should instead be ‘cleansed of dust and quills before the same be put to sale’.^41^ Moreover no one was permitted to ‘put any ffendowne or Thistledown into any wares whatsoever’, and no bedding should be ‘filled or stuffed with wooll, strick haire, Neatshair, Coneywooll [rabbit fur], thistledowne, Hatmakers stuff, cotton or any other corrupt or deceitfull stuff but only with downe, feathers or flocks’.^42^ The bylaws stated that such actions would be punished with a fine of five pounds, an extremely high sum that exceeded the value of most high-quality feather beds.

As a result of the company’s incorporation, regular searches were undertaken in which bedding items found to be stuffed with improper materials were confiscated and sellers were fined. William Gore’s beds were discovered on one such search. These searches were recorded in the company’s court minute books, which give details of the items, the stuffing they contained and the fines issued. While the records do not describe how the searches proceeded, it appears that searchers targeted particular areas of the city, such as Drury Lane, Monmouth Street and Ditchside near Fleetbridge (referred to as ‘Fleet Ditch’ in the records) as mentioned by Thomas Brown, where they knew beds were being sold. Each seller’s wares would then be sampled, with the external ticking or cover of an item being opened to reveal its contents for evaluation. The senses played a key part in this assessment, as the appearance, smell and feel of the stuffing material needed to be ascertained in line with the regulations. While the bylaws suggested that every offence would be subject to a fine of five pounds, in practice fines were wide-ranging, depending on the number of items in question, the kind of stuffing material used, as well as the wealth and profession of the seller.^43^ Upholsterers themselves were something of a minority among those receiving fines, reinforcing the view that the company was targeting sellers operating outside the guild. Most often the occupations of those appearing in court were not stated, but those of slop seller, broker and joiner appear repeatedly. Joiners were woodworkers and furniture makers, some of whom may have been part of the Worshipful Company of Joiners and Ceilers, and may have been viewed as infringing on the upholders’ domain. When upholsterers did appear in court, they were more likely to receive high fines from the company, while slop sellers or brokers would often face a lesser punishment due to claims of poverty or ignorance of the regulations. First offenders were also treated more leniently. Many of those receiving fines were women, several of whom were described as widows, and their presence in these sources, alongside the ‘very poor’, indicates the diversity of those who participated in London’s growing consumer culture in this period.^44^

While searches may have taken place from the beginning of the company’s incorporation in 1626, since most of the company’s records were destroyed in the Great Fire of London in 1666, the earliest court minute book containing details of searches dates from 1678.^45^ This manuscript indicates that between 1679 and 1723 at least twenty searches were conducted that resulted in court sessions. These were roughly every few years with some periods of more intense searches such as between 1700 and 1706, when searches were conducted every summer. The bed filling most commonly confiscated was a mixture of flocks and feathers, which accounted for 30 per cent of the fines issued. It is unclear what exactly was ‘contagious’ about mixing these two acceptable materials, and in several cases those found to have bedding stuffed with both flocks and feathers would be fined but would then receive the items back on the condition they ‘devide the flocks & feathers’, suggesting that despite having been mixed neither material was unsuitable for future use.^46^ The second most frequently confiscated filling was ‘trash’, which probably meant a mix of waste materials including paper, cloth, hair and plant matter. The third and fourth most frequently mentioned materials were ‘squib’, possibly referring to the feathers of a young pigeon or ‘squab’, and coney wool (rabbit fur).^47^ These materials also appear in various combinations with one another and with other prohibited stuffing matter. Other fillings mentioned include thrumbs (loose tailor’s threads), tow (coarse fibres from processing hemp or flax), rags, cotton, dog hair, goat hair, pigeon feathers and thistledown. Several of these items appear in only one or two entries, suggesting that they were less commonly sold, but nevertheless offering a glimpse into the wide range of bedding materials in use in early modern England.

From the various legal restrictions issued between 1474 and 1679, as well as the court documents dating between 1679 and 1723, we gain a richer understanding of the various things people used to fill beds, pillows and quilts across a 250-year period. The repeated issuing of the statutes, as well as the fines recorded by the company, indicate that people continued to sleep on prohibited materials despite repeated warnings about their health implications. While this may reflect the continuing unscrupulousness of upholsterers, the sources also suggest that customers were often aware of the contents of the bedding they bought, providing evidence of a disjunction between health prescription and practice as people deliberately chose beds that authorities deemed unhealthy. Such decisions were made on economic grounds, yet comfort and prestige could also be prioritized above the perceived healthfulness of particular materials as we shall see. From the upholsterers’ records, three broad groups of ‘improper’ bedding materials may be identified, namely, unclean or corrupt feathers, various types of animal hair and wool, and plant matter. The following three sections are structured around these three categories, as I explore how the use (or misuse) of these materials in early modern bedding was connected to questions of health, social status and environment.

*

The regulations of the Company of Upholders suggest that the quality of feathers used in bedding, as well as their impact on the health of sleepers, depended on the methods by which they were removed from the bird’s body. It was repeatedly stated that ‘scalded’ feathers were ‘corrupt’ and should not be mixed with ‘dry-pulled’ feathers, as this was ‘contagious for Man’s Body to lie on’.^48^ By ‘scalded’, the statutes refer to the practice of removing a bird’s feathers by submerging the bird’s carcass in hot water to loosen the feathers from their follicles.^49^ In Hannah Glasse’s *The Art of Cookery* from 1747, she wrote of feather removal, ‘Let your water be scalding hot, and dip in your Goose for a Minute, then all the Feathers will come off clean’.^50^ While this practice was recommended for cooking birds, it was not believed to produce the best feathers for bedding. This may have been connected to the understanding, expressed by French priest Noël Chomel in his *Family Dictionary*, translated into English in 1725, that ‘the feathers of a dead Goose are not so good as those of a live one’.^51^ Naturally, scalding was a practice that required the bird to be dead. The feathers of a live bird were preferable, presumably because they were less likely to be ‘corrupt’ as they did not originate from the carcass of a dead animal. The limitations of scalded feathers for bedding were probably also connected to the damaging effect of scalding on the material qualities of feathers through exposure to intense heat and moisture.^52^ Contemporary naturalists noted, moreover, that healthy birds would oil their feathers to prevent them from becoming wet, thus retaining their warmth and buoyancy.^53^ Wet feathers not only lacked these qualities but could be an indication of sickness in a live bird that failed to properly oil its plumage. Such associations would have contributed to a preference for ‘dry-pulled’ feathers for bedding, which were plucked from the bird while it was still alive.

Such plucking required knowledge of seasonal changes that occurred in a bird’s plumage, including their moulting period, which determined the best times at which to remove feathers. The agricultural writer Leonard Mascall, for instance, commented on ‘what time to pluck’ goose feathers, in his text on poultry from 1581. He stated that many housewives ‘yearely … take a fleece of their Geese, as men doe of their sheepe, which feathers commonlye they take in Iuly and August’. Yet, he wrote,

in some places they take their Geese feathers twise a yeare, in marche they clip all saue their bellies, to couer their yong, & they plucke in August, and some doe plucke in March, and sayeth their feathers will come the sooner or rather then when they are clipte, for in clipping the quills remayne still till melting time, wherefore plucking them is rather counted better [then clipping] to haue two fleeses a yere.^54^

Mascall reflected on the processes of removing feathers, suggesting that simply clipping feathers in March would not provide as good a fleece than plucking the birds in both spring and summer. We also see from Mascall’s statements how the gathering of animal materials could be divided along gender lines, as women are stated to pluck goose feathers, while men shear sheep, perhaps due to the rearing of geese for use in the kitchen, suggesting a greater association with housewifely duties. That one needed to leave feathers on the stomach of geese in spring for the care of their young further indicates how the animal’s own needs, in conjunction with human concerns for their effective reproduction, could shape the practice of gathering feathers, thus requiring collaboration between human and animal.^55^ Mascall explicitly connected such methods of collecting feathers to the stuffing of beds when he stated that with ‘two fleeses a yere’, ‘yee maye vse them as ye shall see cause, to haue their fethers for a more profite to furnish yearely your beddes which are occupied dayly, as in Innes and such’. He suggested that the greater provision of feathers from two periods of plucking thus enabled the regular replenishment of filling for beds that had frequent use and a greater turnover of occupants.

Practices of plucking geese could not be replicated in all environments, however. According to Mascall, ‘Whereas there are greate pondes or riuers nye your house, it is great danger to pluck the feathers of their bellies, for whereas such cold waters are, it is a danger to kill them’.^56^ How one plucked a goose thus depended on the surrounding landscape. Richard Bradley’s translation of Chomel’s text also includes a consideration of temperature. Commenting on goose plucking in France, Bradley wrote, ‘They pluck them the first Time when two Months old, and for the second it is done always, says M. Chomel, in the beginning of November, but with more moderation, because of the Approach of cold Weather, which will make them catch cold’. However, Bradley stated, ‘this Method of pulling the Feathers of Geese twice in the Year, may perhaps do in France; but our best English Authors … assert it to be a very ill way’. This assessment was based on the fact that such plucking made it harder for the geese to fly, putting them at risk from predators, and ‘by unclothing her in Winter you strike that cold into her Belly that kills her suddenly, therefore it is the best Way to stay till moulting Time’.^57^ The best-quality feathers were not available all year round, therefore, but were subject to the conditions of a particular landscape, seasonal temperatures as well as cyclical changes in plumage in response to climatic factors. The collection of feathers could also depend on the migratory patterns of birds, as John Macky described in relation to Solan geese, who would breed on the Island of ‘Ailsey’ (probably Ailsa Craig) in western Scotland. He stated that inhabitants of the island would prepare geese nesting areas before their arrival in July and August. During the birds’ short stay, islanders profited from their collection of fish, as well as their meat, and would ‘stuff their beds with their feathers’, before the geese left the island in September.^58^

The range of variables involved in the collection and use of feathers for bedding is especially evident in contemporary discussions of eiderdown. A description of the Faroe Islands, published in English in 1676, stated of the eider duck that ‘from this Fowle is gotten Eider down which the Eider plucks off from its Breast, and layeth in its nest about the Eggs’. Once the birds left the nest, ‘this Downe is taken up … being then full of Moss and Straw; wherefore it is dryed and cleansed over a basket; the Down which is pluckt off at other times from the Eider is good for nothing, for it is fat and rotteth’.^59^ The collection of eiderdown for human bedding thus depended on the birds’ use of the material to warm their own sleeping quarters, as they lined their nests with down to insulate their eggs. To ensure its sought-after qualities of softness and warmth, the down needed to be removed by the bird itself, thus indicating the animal’s agency in the preparation of this material. This point was emphasized by Gideon Pierreville, general secretary to the king’s minister at the court of Denmark, who wrote, ‘If the feathers be pull’d off by man, they rot away forthwith’.^60^ Once collected, eiderdown was a prized bedding material. The ornithologist Francis Willoughby wrote that ‘Eider dun’ feathers were ‘very soft, and fit to stuff Beds and Quilts. For in a small quantity they dilate themselves much (being very springy) and warm the body above any others. These Birds are wont at set times to moult their feathers, enriching the Fowlers with this desirable merchandize’.^61^ Again, Willoughby noted that eiderdown could be collected only at specific times while indicating the material qualities that made eiderdown particularly suitable for bedding.

Down feathers provided a more costly filling for bedding items than regular feathers due to their fineness and softness.^62^ Sourcing the right kind of feathers for bedding meant understanding the different components of a bird’s covering, including down, feathers and quills. Knowing which kinds of material could be sourced from which birds was important. Richard Bradley stated that down is ‘only found upon what I call Fowls’, of which ‘land fowls … only have this Down upon them till their feathers appear’, while ‘Water Fowls … have always a coat of Down under or beneath their Feathers’.^63^ Moreover, the feathers and down of different species of bird could vary in their suitability for bedding. The feathers of waterfowl, such as duck and teal, were described by Mascall as ‘best to be rather put in cushions then in beds, because they are more hotter, and are a greater soker of men’.^64^ Mascall suggested that the heat of these birds’ feathers would cause one to overheat and sweat in beds that were stuffed with them. The idea that feathers could cause overheating was vigorously presented by London merchant and popular lifestyle author Thomas Tryon, who stated that all feathers were ‘of a strong, hot, fulsom quality’.^65^ Tryon wrote that when ‘a Man is hot and sweats’ in an old feather bed, ‘evil vapours and spirits’ would arise from the feathers that could penetrate the body through its opened pores, disrupting the delicate balance of the body’s humours. He too identified the feathers of fowl to be especially likely to cause sweating, since they ‘of all Creatures, are for the most part of the hottest’. The heat of such feathers would transfer to the body of the sleeper, having a detrimental effect on their health by drawing natural strength away from the organs, disordering the nerves and weakening both body and mind.^66^

Mascall outlined a hierarchy of feathers to be used in bedding, stating that ‘Of all feathers the swanne is the cheefest, and the feathers of the white Goose next, the black or gray Goose feather next him and the Capon and pullet next, likewise all other lande fowle, then Ducke, Wegin, Teale’.^67^ The majority of references to feather beds, in both inventories and upholsterers’ records, do not mention the breed of bird from which the feathers originated. Higher-quality feather beds would in all likelihood have been made of goose or duck feathers, since the curved plumes of these birds provide better insulation, while coarser, flatter feathers such as those of chickens may have been present in cheaper feather beds.^68^

Many such feathers would probably have been sourced as a by-product from poultry producers, for whom John Worlidge stated in 1675, ‘The feathers must needs yield a considerable advantage’.^69^ In such cases, however, feathers would have been plucked from the carcasses of dead birds, suggesting that upholsterers favoured the feathers of birds that had been bred specifically for their plumage.

Pigeon feathers were explicitly stated to be particularly dangerous for stuffing beds and pillows. Mascall wrote, ‘The Pigion [is the] worste feather of all, for they soke too much to be put in beds, and are unwholesome, for they say if a sick person lye on a pillow of pigions feathers, he shall long continue without departing’.^70^ The belief that lying on pigeon feather bedding would prolong one’s suffering during serious illness, preventing one from dying well, continued throughout the seventeenth and eighteenth centuries, though it was frequently challenged. In 1708, for instance, a reader of the *British Apollo* magazine wrote in to ask why pigeon feathers might have this effect. In response, the author stated, ‘This is an old woman’s story. But the scent of Pigeons’ feathers are so strong, that they are not fit to make Beds with’, and so the ‘Nausiousness of the smell has introduc’d a disuse of Pigeons Feathers to make Beds’.^71^ In the *Ladies Diary* from 1791, the idea that pigeon feathers would prolong death was described as a superstition, even a ‘popish ignorance’, and the reason why pigeon feathers were not used to make beds was stated to be because ‘pigeon feathers are usually plucked from the fowl while young, and, as such cannot be fit to make beds of, being mostly pens full of blood, which by drying must become hard and uncomfortable’.^72^ ‘Squib’, one of the most frequently listed materials in the upholsterer’s court records, receiving the third largest proportion of fines, was presumably used to refer to these feathers plucked from young pigeons, which were commonly known as ‘squabs’. The common presence of blood in such feathers may also explain Mascall’s condemnation of them as he stated, ‘all sicke feathers or bloodye feathers are not good to be put in beds, nor Coshions, because they doe commonlye breede wormes’, indicating that such blood would result in putrefaction.^73^ While the feathers of young pigeons could contain blood, older pigeons were considered particularly dry, hard and hot following a lifetime of flight, meaning their feathers were equally unsuitable for bedding.^74^ In addition to health concerns, the disuse of pigeon feathers may have been connected to their association with elites as, throughout this period, pigeons were protected birds that were housed mainly in lordly dovecotes and would have been off-limits to most people.^75^

The type of feather, as well as methods of plucking, determined their suitability for bedding. Unsurprisingly, therefore, these factors were regulated by the London Company of Upholders. Not only should feathers be ‘dry pulled’, but pigeon feathers were specifically prohibited by the company in 1626. The court minute books reveal that fines were issued to individuals who sold bedding containing pigeon feathers. In 1716, for instance, Anne Anderson of Ratcliffe was fined two shillings and six pence for ‘having two pillows filled with pigeons’ feathers’.^76^ The cleanliness and freshness of feathers was also a concern, and the sale of unclean, bad or old feathers would result in a penalty. In 1679, for example, Abraham Trunkett was fined ten shillings for ‘having a bed and bolster filled with feathers not clensed from sand and dust’.^77^ In August 1693 ‘widow White’ was fined five shillings ‘for having one old bed with bad feathers’, while in 1700 Mrs. Perry of Houndsditch was found to have ‘one old Bolster filled with Dross feathers’, but as it was her first offence she was excused and put two shillings and six pence into the poor box.^78^ None of these individuals were members of the company but they were found to be selling bedding items, probably including second-hand bedding, which contained improper materials.

Cleaning feathers was an essential part of preparing them for use in bedding. This involved separating the soft down and removing the quills. The heraldic painter Randle Holme outlined these processes in his *Academy of Armory* from 1688. ‘Dressing’ of feathers, he stated meant ‘making [them] all clean from quills’, the hard part of the feather. This could be done via ‘stripping’ or ‘clipping’, which involved ‘cutting … the feather part from the Quill with scissars’. ‘Fanning’ or ‘driving’ meant ‘taking the down away’.^79^ It was important that the quills were removed, not only because they would be hard and uncomfortable, but also because they might contain traces of the bird’s blood or other excrement. Tryon stated that feather beds ‘possibly stunk before ever they were lain on, by reason of the fulsome excrements that the quills of the feathers contain’.^80^ The material processing of feathers was thus an essential step for ensuring healthy sleep on a feather bed.

The unwholesomeness of old feathers, which had been previously laid upon, was particularly worrying for Tryon, as such feathers would contain the ‘vapours’ of the previous sleeper. This was concerning since English beds, he suggested, would often be filled with ‘feathers that are imported from several countries, which are the drivings of old beds, the uncleanness whereof is not considered’.^81^ Writing in 1682, Tryon echoed a concern that had come to the attention of William Cecil (known as Lord Burghley), chief advisor to Elizabeth I, a century earlier. In 1585 Peter Osborne, a remembrancer in the exchequer, reported to Burghley that he had undertaken an investigation into the practices of upholsterers. Under the pretence of requiring ‘bed stuffe for my howse in the country’, Osborne had spoken with an upholsterer who revealed that

for this vii years last past most feathers … com out of the low countries so packed up out of the spoyle of howses with lyme, durte, dust, stones & all heavy rubbysshe to make them waye, that when they did drive a sacke to make beddes of them they did garboile out … the halfe of souche baggage & refuse stuffe & yet the feathers remained still unmade cleane to the purpose the lyme wold cleave so fast on them.^82^

The upholsterer suggested, therefore, that any searches of bedding materials needed to begin with ‘the merchant that brought it in’, rather than with the upholsterers themselves. If greater inspections were applied to the latter, ‘all retailers were then vndone for all theire stuffe of that kind’ as they could get no other. While nothing came of Osborne’s report, this was worrying intelligence. The issue at stake was not merely that of fraud, as feather beds were made to weigh more through the addition of ‘refuse stuffe’, but also the lime, dirt and dust that such old feathers contained would impact the bodies and health of those who slept upon them. Tryon stated that old feathers caused illnesses in sleepers’ bodies, as ‘by lying upon or in such Beds’, distempers would ‘secretly steal on a Man by degrees, so that he cannot imagine whence the disorder proceeds’.^83^ Lime in particular was a substance known to have a ‘harsh, fiery, keen, sharp, corroding quality’, which could be irritating to the skin.^84^ English naturalist Robert Lovell explicitly warned against the use of lime to whiten linen because this ‘biteth and causeth scabbiness’.^85^

Lord Burghley was appealed to again on the subject of upholsterers when, in 1594, a group of upholsterers wrote to defend themselves against accusations of ‘fraud in feathers’. They noted that ‘as touching feathers they are of many sortes and in their owne Nature do much varie’. They continued by stating that ‘it is not possible to make those good which themselves are bad and if they do once smell they can not be made sweet except It be with some continuance of wearing’. Acknowledging the difficulties of working with feathers, which could be bad or smelly, they also minimized their own responsibility for the quality of the feathers their beds contained, writing that any dust contained in feathers ‘is by reason of the sandiness and drines of the soile wherein they are bred’ and could not be helped.^86^ They indicated, therefore, how a bird’s environment was understood to influence the quality of the feathers they produced. The upholsterers also commented on the importation of feathers and connected this to practices of plucking. They stated, ‘There is but one countrie which is France from whence they come where they do once a yeare plucke their geese alive and let them goe againe and this is the cause that no other fethers being now will be sweet which French fethers are’. While the dry plucking of live birds was thought to result in superior feathers, the company thus suggested that such feathers could only be imported from France. In contrast to Chomel, they suggested that the birds would be plucked only once a year, meaning there would be ‘few [feathers] if we had no other’, justifying their use of lesser-quality feathers where such ‘sweet’ French feathers could not be sourced.

A month later a separate group of London upholsterers challenged this petition to Burghley. They stated that the sandiness of some feathers was due to merchants who ‘putt greate quantitie of sande, dust and quills amongst them to make them more waightie’ and ‘doth not come by reason of the sandyness and dryness of the soyle’.^87^ In this exchange we see a tension that was present throughout discussions of prohibited bedding materials, which centred on both the deceit of bed sellers and the nature of the materials contained in the beds they sold. The latter was recognized to depend on practices of obtaining and preparing the material, the nature of the animal or plant from which it originated and the environments in which they lived and grew. This tension also shaped discussions concerning animal hair, the second category of bed stuffing materials to be considered.

*

Like feathers, animal hair could be problematic as a bed stuffing material, partly because it was believed to possess the qualities of the animal from which it originated. Writing of the use of animal hair as an organic fertilizer for crops, agricultural writer William Ellis wrote that ‘both cows and Hogs-hair are certainly of the same Nature with the Blood, Flesh and Hide of the Creatures they are taken from; and I presume, this Nature, or Quality, is of the oily and sulphureous Kind’.^88^ Such properties made cow and pig hair an ideal fertilizer but an unsuitable bedding material.^89^ This was equally true of wool. Though the 1551 statute of the Company of Upholsterers stated that permissible bed stuff consisted of ‘Feathers, Wool or Flocks alone’, by 1679 the company’s bylaws insisted that no bedding should be ‘filled or stuffed with wooll’.^90^ This did not apply to woollen flocks that had been processed, but to wool in its raw form, the dangers of which were outlined by Joshua Smith, a supplier of flocks to the Royal Navy, in 1705. Smith stated that ‘Wooll in its first nature is foole in it selfe by Reason it is taken of with Lime’. Caustic lime was a known irritant that was understood to be hot and corrosive. According to Smith, raw wool also contained a residue ‘of the Skinn and the grease and salve that is in it and in hott wether is noisome and infectious and not Proper for [use in bedding] and if washt and not well dried is noysome as to the smell and Destruktive to mens Bodies to ly upon’.^91^

That the ‘infectious’ qualities of raw wool were elevated in hot weather is of particular significance in Smith’s correspondence, as the flocks with which he proposed to supply the navy would be used in bedding on ships that might sail to hot climates, where raw wool would be a particularly impractical and unhealthy stuffing option. Such concerns about the healthfulness of wool in hot weather are also likely to have been connected to contemporary medical teachings underlining the importance of a cool environment for healthy sleep.^92^ To prevent infection, Smith proposed flocks of woven woollen cloth that would then go through a process of fulling, by which the material was cleaned and thickened. Though ‘Wooll may be Bought for almost halfe the Price of flocks’, Smith insisted that beds stuffed with flocks ‘are worth halfe the money after they are worne three or four years’, whereas beds filled with raw wool, ‘are worth nothing at all after they are worne two or three years but to throw upon the Dunghil’. Smith thus presented the shorter lifecycle of the cheaper bed filling to decrease its appeal to navy officials, at the same time indicating how the beds of poorer communities, who could not afford better-quality bed fillings, would require more regular replacement. To be suitable for bedding, especially in hot climates, woollen flocks needed to be properly cleaned and processed, indicating the conjoining of material and environmental knowledge for stuffing beds, a practice that was further dictated by factors of wealth and class.

Beds containing wool that had not been properly cleaned would result in penalties in the upholsterer’s court. In 1702, for example, an upholsterer named James Eckley was fined for having ‘one calico Quilt filled with bad wooll’, while in 1704 William Browne of Tower Hill was charged for having a seabed ‘filled with uncleane wooll’.^93^ The bed was later returned to Browne, however, on the condition that he would ‘cleane the wooll’, suggesting the condition of the material was more significant than the use of wool itself. It could also matter which area of the sheep’s body the wool came from as the use of shank wool, from a sheep’s leg, was punished with a fine. John Cockburn paid a pound for having one bed filled with shank wool in 1721, while Nathaniel Goadby paid five shillings for two bolsters stuffed with it in 1723.^94^ This was probably due to shank wool being considered coarser and less clean than wool from the main fleece.

The quality of the wool could also depend on the nature of a sheep’s pasture. Edward Topsell noted that feeding sheep ‘in fat pastures’ would cause them to have ‘softer wool or hair, according to the nature of their food’, and he stated, ‘Because they are of a moist temperament, it is better to feed them upon the salt and short pasture: for by such a diet, they both live in health, and also bear more pretious wooll’.^95^ Here we see how the health of the animal related to the quality of material it produced, which would in turn affect the health of the human bodies with which it came into contact. Topsell related this specifically to particular regions of England, writing that ‘the whole county of Buckingham is of a clammy, champain, fertile soil, feeding innumerable flocks of sheep with his rich and well-growen pastures, whose soft and fine fleeces of wool’ were particularly desirable.^96^ Climate would determine the time of year when sheep were sheared, as Topsell wrote: ‘In some hot Countries they shear their Sheep in *April*, in temperate Countries they shear them in *May*, but in the cold Countries in *June* and *July*’, since such sheep would need their fleeces for longer. Methods of shearing sheep were also known to influence the quality of their fleece, as Topsell stated that some would shear them twice in a year ‘for the opinion that the often shearing causeth the finer wool to arise’.^97^ As for feathers, the care of the animal and its habitat was recognized to influence the quality of the material it produced. While these factors are not explicitly referenced in the company’s records, their implications for the supply of good-quality wool would have made them issues of concern for those buying and selling beds stuffed with wool or flocks.

In addition to bad or unclean wool, the regulations of the Company of Upholders reveal a wide range of animals whose hair was forbidden for use in bedding. These included horse, cow, deer, goat, rabbit and ‘stricke’ referring to a young bullock or heifer. The specific naming of these animals in the sources indicates that bedding stuffed with such hair had been used or discovered at some point. The upholsterer’s court records also reveal that animal hair was a popular type of bed filling, since coney, goat and dog hair repeatedly appear among the materials resulting in fines at the upholsterer’s court. The frequency of animal hair found on the company’s searches may have stemmed from its desirability. In the 1594 petition, for instance, members of the company responded to the accusation that they sold quilts stuffed with cow hair. They wrote that they did not sell such items, ‘although they are much desired of [by] many and especially of Innkeepers who know that they are stuffed with cow haire yet they can fynd nothing for that price to serve their purpose nor so well to content their guestes as they saie’.^98^ While their opponents challenged this view, stating, ‘It is not lyklie that Inkepers doe desire them, but if it be soe there guests have little cause to thanke them for there lodging’, the exchange suggests why such prohibited materials might be favoured by some.^99^ The attraction of cow hair for innkeepers is shown to be its low cost and durability, as it could sustain a regular turnover of guests while remaining relatively comfortable.

In some cases, animal hair could even be deemed to be a healthful bedding material. In the 1664 English edition of Felix Platter’s *Golden Practice of Physick*, for example, he stated that if one suffers from ‘pain of Hypochrondria, or sides under the Ribs’, ‘the actual Heat of bags made of dryed simples, of Chaff or Mililum is good here’, as are ‘pillows and skins, or Furrs, Coney-wool and hares skins are the best’.^100^ Platter thus advised using a pillow stuffed with coney wool, meaning rabbit fur, to provide heat that could be applied to the area in pain.

Conies were often described in terms of their heat, which accounted for their lustful nature; as Gervase Markham stated, ‘They are violently hot in the act of generation’.^101^ The perceived heat of conies probably informed the desirability of coney wool as a bedding material, for comfort and warmth, as well as its prohibition by the Company of Upholders, since hot bedding could prevent healthy sleep. Coney wool is a prime example, therefore, of the distinction between prescription and practice that appears in these records. Though it was prohibited, coney wool items appear in every major search conducted by the company between 1679 and 1723, and coney wool was the fourth most fined material in the upholsterer’s court records. In 1700, for example, Ann Bayly from Barbican was fined two shillings and six pence for having an old bed of coney hair and two pillows filled with flocks and feathers for sale.^102^ In 1705 Robert Shreeve of Drury Lane was found to have ‘one old bed, bolster and Pillow filled with coney hair’. His wife appeared for him in court as Mr. Shreeve was ‘sick in the country’, paying a reduced fine of five shillings due to their ‘being poor’.^103^ Some years earlier, in 1682, an upholsterer named Jonathan Everard near Drury Lane was found to have a ‘cunny wool bed’, yet he appeared in court with a Mr. Carter, ‘who did declare the Bed was his proper goods and by a mistake was sent to the said Edwards House’.^104^ The court noted that this account appeared ‘plaine, as likewise the testimony of a waterman that caryed the said Bed to Everards’, and thus ‘this court thought good to order the said Bed to be returned to the said Carter the owner of it’. This is an important reminder that it was only beds that were to be sold that were not permitted to contain such materials, though it is unclear where Mr. Carter himself sourced his coney wool bed. Nevertheless, he clearly wanted to retain the item, suggesting that this bed, including its filling, was something he prized.

Coney wool beds and bedding could be costly, valuable items. The Bristol hair weaver Thomas Compton’s probate inventory from 1690 shows that he owned two ‘coney fur’ beds, which, together with their accompanying feather bolsters, were worth one pound and one pound and five shillings, respectively.^105^ This was not significantly less than a feather bed, depending on quality and weight. The probate inventory of Robert Lux, a Bristol wool-comber who died a year later than Compton, shows that his feather bed was valued at one pound and fifteen shillings, while Lux’s flock bed and quilt was worth only fifteen shillings.^106^ When John Grisse, a yeoman from Salem County, died in 1688, he left behind a ‘rabbit fur bed and bedding’ worth five pounds, while the whole value of his forty-acre farm was listed at twenty pounds.^107^ The inflated value of Grisse’s rabbit fur bedding may have stemmed from its relative rarity in the American colonies, yet in England too, coney wool was a valuable commodity that was in high demand due to its use in hat making. Writing about methods of increasing the wealth to be gained from the colony in Virginia, Samuel Hartlib suggested the ‘multiplying of cunny warrens’, which he deemed to be ‘so easie a thing’ and with great reward, since ‘the wool of a skin is now worth 8 pence, which is more then the body’ and ‘will be very vendible’ for creating hats and stockings.^108^

Due to its high value, thefts of coney skins and fur repeatedly appear in the records of the Old Bailey. In 1682, for instance, Ann Jaxon was indicted for stealing coney skins ‘to the value of 7 pounds, from a Furrier’, while in 1712 Elizabeth Isham and Sarah Cullam were indicted for stealing twenty pounds of coney wool valued at ten pounds, from the warehouse of Benjamin Mason.^109^ Richard Williams faced transportation for ‘stealing a Coney-wool Bed, a pair of sheets, and other things’ in 1726.^110^ That Williams specifically stole a coney wool bed supports the view that this type of bed filling could be particularly desirable. In 1682 the Company of Upholders seized two beds and a bolster filled with ‘Cunney hair’ from upholsterer Edward Blanch. To fulfil the demands of the 1551 statute, in November of the following year it was ‘ordered that one of the Couney hair beds [which was formerly seized in the house of Edward Blanch] be delivered to the Bayliffe of the Burrow of Southwarke being the moyety due to the Kings Ma[jes]tie’.^111^ The use of the coney wool bed to pay the demanded moiety to the king further highlights the value, and even prestige, it was acknowledged to hold despite being a material forbidden by the company.

Demand for coney wool was so high in the late seventeenth century, that the Company of Felt-Makers petitioned to prevent its exportation.^112^ The Company of Skinners opposed this, insisting that such an act would ‘abate the yearly value of the coney warrens of all the nobility and Gentry of England, and be an unexpressible loss to many Hundred poor Families who … thereby live very comfortably’.^113^ As the Skinners indicated, rabbit warrens were lucrative sources of income, primarily for elite landowners, whose warren rights granted them the exclusive privilege of keeping and killing rabbits, while the preparation of meat and furs provided income for surrounding towns and villages.^114^ Like pigeons, rabbits were prized commodities that symbolized elite status and privilege in this period.^115^ As the English merchant and agricultural writer John Mortimer wrote in 1708, rabbits were ‘very profitable Creatures’ for landowners because they could be ‘kept on dry barren Sand or Gravel that will maintain nothing else’.^116^ Regions of poor soil thus became considerable sources of wealth through rabbit rearing.^117^

Though official access to rabbits remained restricted, the sixteenth century witnessed a dramatic increase in the population of rabbits, which often strayed beyond the boundaries of their warrens, destroying crops on common land and fuelling tensions between landowners, commoners and tenants.^118^ Poaching rabbits became a form of protest against elite privileges.^119^ An act concerning the preservation of conies in Lincoln from 1765 stated that all those ‘convicted of entering warrens in the night-time, taking or killing conies there’ would be subject to transportation for seven years, or face whipping, fine or imprisonment. However, the act made clear that such punishment did not ‘extend to the destroying of conies in the day time … upon account of the great mischief and damage occasioned by the increase of conies upon the fen and river banks in the said county’.^120^ Coney wool first appears as a prohibited material for bedding in the 1679 bylaws of the upholsterers’ company, probably due to the increasing accessibility of rabbits, which, by that time, could more easily be found outside their warrens. To have a coney wool bed for sale, however, would nevertheless raise questions about where the stuffing material had been sourced, as the killing of conies remained tied to elite privileges and their fur was typically the preserve of furriers and felt makers. Despite these restrictions, the continued making of beds with coney wool indicates not only that salespeople were able to source this material but that there was demand for these items. Coney wool bedding was prized for its warmth, but its desirability must also be understood within the context of elite warren rights and the high cost of rabbit fur and skin. Rabbits’ value was connected to their ability to transform barren land into a source of profit and wealth. This carried important implications for regions of poor soil and further indicates how questions of environment and landscape were intertwined with early modern sleeping arrangements.

*

Environment and landscape were of critical concern for the use of plant matter to fill beds, which depended on the availability of plant species within a particular region. In his *Pambotanologia* from 1659, for instance, Robert Lovell wrote of mat weed grown in Spain and the Low Countries, of which ‘the soft [varieties] serve to stuff beds with’.^121^ Travel accounts repeatedly commented on the use of indigenous plants for filling beds and pillows. In a text translated into English in 1669, Adam Olearius reported that in the Azores Island there was a plant that grew five or six feet high and was attached to the earth by very fine roots, ‘as small as the hair of a Man’s head’. These roots were used by local people ‘instead of wooll and feathers, to fill their Beds and mattresses withall’.^122^ In the early eighteenth century Hans Sloane wrote of the cotton tree in Jamaica, stating, ‘They stuff beds with this down … but they are not counted healthy to lye on’.^123^ This assessment of the healthfulness of this bedding was presumably not shared by the local people who used it.

The use of plant materials for bedding also depended on the distinctive ecologies of particular regions. Lovell, for instance, wrote of the common bog plant cotton grass, which required a wet environment to produce ‘woolly heads’ that ‘serve for the stuffing of beds’.^124^ This was echoed by William Salmon, who wrote of cotton grass, which ‘grows in moist, wet, Boggy and Moorish places’. Salmon stated, ‘The Woolly Heads are gathered by some to stuff Pillows, Bolsters, and Cushions with’.^125^ As these plants could be found only in boggy areas, such descriptions indicate a place-based knowledge of botanicals that was used to create comfortable bedding.

Contemporary descriptions of plant bedding frequently assessed its suitability for comfortable sleep. In his discourse on forest trees from 1664, for instance, John Evelyn stated of the beech,

Its very leaves … being gathered about the Fall and somewhat before they are much Frost-bitten, afford the best and easiest Mattresses in the World to lay under our Quilts instead of Straw; because besides their Tenderness and loose lying together, they continue sweet for seven or eight Years long, before which time Straw becomes musty and hard.^126^

Evelyn reported that beech leaves were used for this purpose in south-eastern France and that ‘in Switzerland I have sometimes lain on them to my great Refreshment’. He indicated the seasonal collection of this plant material, which he valued for its softness, durability and longevity. Interestingly, in Evelyn’s extensive diary, his only reference to sleeping on leaves is from northern Italy, where he wrote, ‘In this wretched place, I lay on a bed stuffed with leaves, which made such a crackling and did so prick my skin through the tick, that I could not sleep’.^127^ Evelyn’s experience perhaps confirmed his view that only the leaves of specific trees, such as the beech, were suitable for sleeping upon. His discomfort also appears to have been caused by the lack of any intermediary layer between his body and the leafy bed. In this anecdote, Evelyn draws attention to the sensory aspects of bed stuffing, as the sound of the crackling leaves is shown to have contributed to his poor sleep. The significance of sound for sleep also appears in *Sylva*, where Evelyn mentioned that the leaves of ‘chesnut-trees make very wholesome Mattresses to lie on … But those leafy-beds for the cracking Noise they make when one turns upon them, the French call *Licts de Parliament*’.^128^

A common thread in descriptions of plant-based bedding, especially those used outside England, was the comparison of such items with those made of thistledown, the fluffy seeds of the thistle. The botanist and apothecary John Parkinson, mentioned in his *Theatre of Plants* (1640) that the down of the swallow wort, found in the mountains of France and Italy ‘make a farre softer stuffing for cushions or pillowes or the like, than thistledown, which is much used in some places for the like purposes’.^129^ Similarly Salmon stated that cotton grass ‘far excell[ed] in softness and goodness any Thistle Down’.^130^ While suggesting that such alternative plant fillings were superior, these descriptions indicate that thistledown was a relatively common and well-known bedding material. This is supported by herbal and botanical texts describing the properties of the common thistle. English herbalist John Gerard wrote of the thistle, ‘whereof the greatest quantitie of down is gathered for diverse purposes’ including its collection ‘by the poore to stop pillows, cushions and beds for want of feathers’.^131^ Gerard noted that such thistles grew by ‘high waies sides and in ditches almost euery where’, showing thistledown to be a highly accessible bedding material that could provide a cheap, if not free, bed filler for poorer communities. Though other plant matter was stated to be softer than thistledown, the comfort it provided would have been greater than other cheap bed fillers such as straw or reeds.

That thistledown provided a low-cost alternative to other bed fillings is clear from its occasional inclusion in probate inventories. At his death in 1557 William Auger a Bedell at St Andrew’s Parish in Cambridge left two mattresses of ‘thystle downe’ that were valued at just four pence.^132^ In addition to its use ‘by the poor’, however, Gerard stated that cheap thistledown was used by ‘rich upholsterers to mix with the feathers and downe they do sell’ to deceive their customers.^133^ Gerard thus returns us to the records of the Company of Upholders. From the earliest efforts to regulate bed stuffing in 1474, thistledown was listed among prohibited bedding materials. The company’s court minute books reveal that items containing thistledown were confiscated and their sellers fined.^134^ While the sale of thistledown bedding was consistently forbidden, however, upholsterers had greater concerns about the increasing use of ‘fen down’ for filling beds and pillows.

‘Fen down’ appears as an improper bedding material from 1495. In the earlier petition from 1474 the plant ‘cattes tailes’ was mentioned in its place. In Gerard’s herbal he describes the ‘cat’s taile’ (*typha*, often known as bulrush in British English), which ‘groweth in pooles and such like standing waters and sometimes in running streames’.^135^ This plant, he stated, had a ‘browne knop or eare, soft, thicke, and smooth … which being ripe turneth into a downe and is carried away with the winde’. On the virtues of cattail down, Gerard wrote, ‘This Downe in some places of the Isle of Elie, and the low countries adioyning thereto, is gathered and well sold to make mattresses of, for plowmen and poore people’. Gerard’s statement that the down of the cattail was gathered in the fenlands around Ely to make bedding, suggests that the ‘fen down’ prohibited from 1495 onwards was the same material as the ‘cattes tailes’ listed in 1474. This is corroborated in Salmon’s 1710 *Botanologia*, in which he wrote of the ‘cats-tail’, ‘I have also found them growing in many places in the Fens, and in Moist and Standing Waters in Fenny Grounds in Cambridgeshire, and the Isle of Ely’. He continued, ‘In the Fen Countries [the down] is sometimes used to make Beds of, for poor people to lye on’.^136^

The low cost of fen down presumably explains its relative absence in more traditional sources used to examine early modern bedding materials. Probate inventories, for instance, were selective accounts of household possessions, which focused on items judged to have a significant resale value.^137^ Nevertheless, like thistledown, traces of fen down may occasionally be found in these sources. Scholar’s servant James Hartley of Cambridge, for example, left ‘7 beds of cattall doone’ worth twenty-six shillings in 1601.^138^ Moreover, Richard Meakes, a barber surgeon in Cambridge, had ‘a fen downe bed’ and three fen down bolsters in 1604.^139^ The presence of fen down in inventories from early modern Cambridge probably reflects the regional use of this material in areas surrounding the fens.

Fen down was also transported to London, however, thus reversing the direction of influence that is typically found in examinations of the material culture of the early modern poor, as the regionally specific use of this material was replicated in an urban settlement.^140^ The use of fen down for bedding caused tensions among London upholsterers, who expressed their views on the material in their 1594 petitions to Lord Burghley. Those defending the trade against accusations of fraud, wrote of fen down,

In some cases we find it tolerable, that is for stuffing of cushions and making bedding for the poore whose abilitie may not reach hyer, for we know that the stuffe it selfe will breed nothing that is hurtfull to the health of the bodie onelie in the first yeare it is gathered it will breed a worme while it is greene but being a yeare old the stuff is as sweet and cleane as any fethers and for this order is taken by us that none shall be used until it be a yeare old as also by the parties that gather them which is the poore people in the Yle of Elye.^141^

These upholsterers indicated a material knowledge of this plant matter, which needed to be properly dried or it might ‘breed a worme’. This understanding, which echoes Mascall’s comments concerning bloody feathers, drew on contemporary ideas about putrefaction, based on Aristotle, by which pests were thought to be spontaneously generated from corrupt materials.^142^ According to Tryon, putrefaction in bedding was accelerated through contact with the sleeper’s body, since bodies emitted heat and ‘putrified vapours’ and ‘where there is not heat nor humidity, there can be no putrefaction’.^143^ Fresh fen down, combined with the heat and vapours of the body, would thus result in an extremely unhealthy sleeping environment. Thorough drying of this material was shown to prevent such corruption, knowledge that the people of Ely, who collected the down, were said to share.

Later in their petition, the upholsterers suggested that fen down had replaced animal hair in cheap bedding, and they stated,

Neither can we see that [fen down] can be altogether taken away, the reason is that flockes are of so high a price that the poor can not reach them neither can the quantitie of flockes be gotten in all England to serve for this purpose wherby many poore people shold lye in the straw or full hard and it is used for matrices to lye under a fether bed to keep it from the cordes.^144^

The upholsterers emphasized the continuing demand for fen down among poorer communities, suggesting it was favoured for the comfort it provided, since poor people would otherwise lie on harder bed stuff. They also mention the layering of fen down beds with feather beds, something that is evidenced in Richard Meakes’s inventory, where ‘in the chamber of the hawll’ he had ‘a fether bed’, ‘a fen downe bed’ and two mattresses of unknown filling.^145^ Following this practice, the upholsterers implied, fen down could be acceptable since it would not come into close contact with the body, mitigating the potential for ill health.

In their response, however, the opposing group of upholsterers claimed that ‘fen downe is not tolerable in anye thinge’. They stated,

Yf it be putt in Cusshions or any kinde of bedding before it be 2 yere ould it turns altogeather to wormes and maggotts. Yf it be putt in cushions etc after it is twoe yere ould it turns all to dust and dross and it is to be throwne out to the Dungehill. Yf it be used in flocke beds it filleth mens bodies with most filthie diseases.^146^

These upholsterers rejected the idea that drying fen down would prevent it from putrefying, instead emphasizing the detrimental effect they deemed fen down had on the sleeper’s health. They also rejected their opponents’ argument concerning the availability and cost of fen down in comparison with flocks and feathers. They suggested that the use of fen down was limiting the quantity of feathers imported into London, as merchants ‘doe daylie complayne that there is soe muche fenn downe and thistle downe bought for as a pound that they can have noe vent here for there feathers’. Thus, ‘the greater use of fenn downe, hindereth the cominge over of feathers, because it is so cheape and good feathers can not be souled’. They even argued that a good flock bed could be cheaper than one stuffed with fen down, since ‘sufficyent men shalbe found and bound to serve the whole Realme with large mattrises and flockebedds stuffed with good wooll and good flockes for vi s[hillings] and vii s[hillings] a piece which are nowe solde for x s[hillings] and xii s[hillings] beinge stuffed with fenn downe’. Like for feathers, they claimed ‘there are flocks sufficient’, yet ‘upholsters will not buye [them]’. The influx of fen down in London’s upholstery trade was thus described as being so great that it damaged the supply and demand for flocks and feathers. Finally, this group stated that upholsterers made large profits on beds containing fen down, which they sold ‘to the poore’, since such beds cost them just six pence to make but were sold for ten or twelve shillings. Yet ‘within verie short tyme [the beds] turned all to wormes and it is most filthie and corrupt, and this is the charitable meanes whereby they helpe the poore’. Once again emphasis is placed on the shorter lifecycle of cheaper materials and this was not merely an economic concern, but one grounded in the view that fen down bedding would spread disease among the poor, in turn putting wider society at risk.^147^

Concerns about the use of fen down among the poor may also have drawn on a wider contemporary discourse in which material discomfort, and specifically hard beds, could be viewed as medically and morally superior. John Moneypenny, for example, wrote about people of the Isles of Scotland who rejected feather beds, instead resting on ‘much more wholesome’ bracken, which restored ‘the strength of the sinewes’, causing them to ‘rise in the morning whole and able’.^148^ Similarly, John Locke wrote, ‘Hard lodging strengthens the Parts; whereas being buried every Night in Feathers melts and dissolves the body’.^149^ The robust hardiness associated with material deprivation was specifically connected to the living conditions of the poor, many of whom could not choose otherwise.^150^ Yet the use of cheap fen down as a bedding material enabled some poorer communities to choose comfort over health, a trade-off for which they would have been sorely judged. Since the strength of a nation depended on the physical and moral strength of its people, trading health for comfort was bad for individuals and for the nation. Upholsterers’ comments regarding the increasing use of fen down in beds intended for the poor must, therefore, be viewed against this cultural backdrop, which would likewise have informed the reaction of Lord Burghley to this information.

Fen down caused controversy among upholsterers, who centred their arguments around issues of health, trade, accessibility and deception. Despite their opposing positions, both factions were united in the view that fen down was becoming an increasingly common bedding material at the end of the sixteenth century. It is important to note, however, that flocks and feathers remained by far the most commonly listed bedding material in contemporary inventories, including pauper inventories, and it seems unlikely that fen down posed any real threat to their prominence as bed stuff.^151^ Nevertheless, the upholsterers’ exchange suggests that a trade dedicated to collecting fen down thrived in the early modern fenlands. Such collection would have taken place on the fens’ abundant common waste, where poor inhabitants supplemented their livelihoods by harvesting the natural produce of the wetlands: ‘collecting reeds and sedge for thatching roofs; digging out peat which was dried and burned for fuel’; and, as this article has shown, collecting fen down to sell to London upholsterers.^152^ Eric Ash has demonstrated that despite the fens possessing a reputation for being poor, unhealthy and uncivilized during this period, fenlanders had developed ways to live and prosper in their watery environment. In doing so, they ‘did not simply collect, use and sell whatever the fens had to offer; they worked to shape the landscape, maximizing its advantages and minimizing its inconveniences’.^153^ The collection, sale and use of fen down needs to be understood as part of the dynamic interaction between landscape, ecology and human beings that took place in the fens prior to the widescale drainage projects of the seventeenth century. Fen down’s use in bedding depended on the inhabitants’ knowledge of the local landscape, its cyclical rhythms and its natural vegetation; the very presence of cattails in the fens was owed to the regular flooding they experienced. Fen down thus provides a particularly compelling example of the entanglements between environment, agricultural economies, health and sleep during this period, showing how efforts to sleep well shaped, and were dictated by, local environments.

By 1600 the fens were increasingly perceived by political elites as regions in need of drastic human intervention to increase their productivity and value.^154^ This resulted in a series of drainage projects, beginning in 1625, which had a devastating impact on the traditional ecology and economy of the fens. This included the purposeful replacement of native reeds and rushes in favour of cash crops such as flax, oilseed and hemp. Rushes, like the cattail plant, were described as ‘superfluous and noisome weeds’ the removal of which would increase the profitability of the land.^155^ Roughly a third of common wastes were lost, and fenlanders could no longer rely on traditional by-employments of fishing, fowling or gathering reeds.^156^ Fen drainage must also have impacted on the collection of fen down. Though it was listed among the materials forbidden for use in bedding in the 1679 bylaws of the Company of Upholders, well into the period of drainage projects, fen down did not appear in any of the searches conducted by the company between 1679 and 1723. While other factors may have contributed to this absence, it seems highly likely that the extensive efforts to drain the fens during the seventeenth century, as well as the vilification of rushes and reeds as unprofitable by pro-drainage writers, put an end to its collection on the scale reported in the late sixteenth century. Human and environmental interactions clearly had consequences for sleep, as forced landscape change dictated the availability of this bedding material.

*

The content of one’s bedding mattered in early modern England. The materials with which beds and pillows were stuffed had the potential to generate pests, cause contagion, prolong death and ultimately destroy the bodies of those who slept upon them. It is little wonder, therefore, that bed fillings were highly regulated through acts that sought to prevent the ill health caused by improper bedding and the deceptive practices of upholsterers. Despite such restrictions, however, early modern bed stuff remained diverse, consisting of far more than feathers, flocks and straw. Plant matter, such as thistledown and fen down, provided a cheaper alternative for poorer communities, while coney hair was a popular stuffing for those seeking warmth and comfort despite the elite privileges that restricted access to these animals. The collection of all such materials depended on environmental and material knowledge, as one needed to know in what region, landscape and season they could be sourced, as well as how to clean, dry and prepare them for use in bedding. Efforts to sleep well in early modern England were thus shaped by dynamic interactions between people, plants, animals and environments.

## Footnotes

1 London Metropolitan Archives (hereafter ‘L.M.A.’), Guildhall Library, Worshipful Company of Upholders, MS07141/001, Court Minute Books 1678–1723, p. 165.

2 J. F. Houston, *Featherbedds and Flocks Bedds: the Early History of the Worshipful Company of Upholders of the City of London* (Sandy, 2006); and K. M. Walton, ‘The Worshipful Company of Upholders of the City of London’, *Furniture History*, ix (1973), 41–79.

3 Upholsterers Act 1495, ‘What Stuff Upholsters shall put in Bolsters, Feather-beds and Pillows’ (11 Hen VII, c. 19); and Stuffing of Feather Beds, etc. Act 1551, ‘An Act for stuffing of Feather-Beds, Bolsters, Mattresses and Cushions’ (5 & 6 Edw. VI, c. 23).

4 The National Archives of the U.K., ADM 106/418/18, Alderman Gore, London, 23 Dec. 1692.

5 L.M.A., MS07141/001, p. 171.

6 It seems likely that the unlawful goats’ hair filling would have been removed and disposed of, while the ticking in which it was contained could be resold.

7 L.M.A., MS07141/001, pp. 179–80.

8 M. Berlin, ‘“Broken all in Pieces”: artisans and the regulation of workmanship in early modern London’, in *The Artisan and the European Town, 1500–1900*, ed. G. Crossick (Farnham, 1997), pp. 75–91. On ritualized destruction by the broderers’ company, and its consequences for women makers, see L. Gowing, *Ingenious Trade: Women and Work in Seventeenth-Century London* (Cambridge, 2021), pp. 65–68.

9 Berlin, ‘“Broken all in Pieces”‘, pp. 81–3; and N. Korda, *Labors Lost: Women’s Work and the Early Modern English Stage* (Philadelphia, 2011), p. 178.

10 Upholsterers Act 1495.

11 For contemporary concerns surrounding bedding materials and healthy sleep, see, e.g., Thomas Tryon, *A Treatise of Cleanness in Meats and Drinks* (London, 1682), pp. 5–12; Everard Maynwaring, *The Method and Means of Enjoying Health, Vigour and Long Life* (London, 1683), pp. 146–7; Thomas Cogan, *The Haven of Health* (London, 1612), pp. 235–6; and John Locke, *Some Thoughts Concerning Education* (London, 1693), pp. 23–5.

12 S. Handley, *Sleep in Early Modern England* (New Haven, Conn., 2016), pp. 39–40.

13 S. Handley, ‘Objects, emotions and an early modern bed-sheet’, *History Workshop Journal*, lxxxv (2018), 169–94, at p. 172; and Handley, *Sleep*, esp. pp. 40–61, 94–107, 110–48.

14 See Handley, *Sleep*, pp. 121–31. On the material culture of sleep, see also H. Morgan, *Beds and Chambers in Late Medieval England: Readings, Representations and Realities* (Woodbridge, 2017), pp. 20–39; and A. McShane and J. Begiato, ‘Making beds, making households: the domestic and emotional landscape of the bed in early modern England’ (prepublication draft, accessed 2022).

15 L. Weatherill, *Consumer Behaviour and Material Culture in Britain* (London, 1988), p. 160.

16 Handley, *Sleep*, p. 123.

17 T. Brown, *Amusements Serious and Comical, Calculated for the Meridian of London* (London, 1702), p. 45, quoted in S. Pennell, ‘Making the bed in later Stuart and Georgian England’, in *Selling Textiles in the Long Eighteenth Century: Comparative Perspectives From Western Europe*, ed. J. Stobart and B. Blondé (London, 2014), pp. 30–45, at p. 36.

18 C. Muldrew, *Food, Energy and the Creation of Industriousness* (Cambridge, 2011), pp. 195–6.

19 Pennell, ‘Making the bed’; and L. Gowing, ‘The twinkling of a bedstaff: recovering the social life of English beds 1500–1700’, *Home Cultures*, xi (2014), 275–304.

20 J. Harley, ‘Consumption and poverty in the homes of the English poor, c. 1670–1834’, *Social History*, xliii (2018), 81–104, at p. 192; and T. Murdoch, ‘Fit for a king: the state bed from Melville House, Fife’, *Apollo*, clv (2002), 3–9.

21 J. Harley, ‘Consumption and material culture of poverty in early-modern Europe, c. 1450–1800’, in *The Routledge History of Poverty, c. 1450–1800*, ed. D. Hitchcock and J. McClure (Abingdon, 2020), pp. 185–205; Muldrew, *Food*, esp. pp. 163–207; C. Shammas, *The Pre-industrial Consumer in England and America* (Oxford, 1990); B. Lemire, *Global Trade and the Transformation of Consumer Cultures* (Cambridge, 1990); and J. Thirsk, *Economic Policy and Projects: the Development of a Consumer Society in Early Modern England* (Oxford, 1978).

22 J. Styles, ‘Lodging at the Old Bailey: lodgings and their furnishings in eighteenth-century London’, in *Gender, Taste, and Material Culture in Britain and North America, 1700–1830*, ed. J. Styles and A. Vickery (New Haven, Conn., 2006), pp. 61–80.

23 Harley, ‘Consumption and poverty’; and J. Harley, ‘Domestic production and consumption in poor English households, 1670–1840’, *Agricultural History Review*, lxix (2021), 25–49.

24 M. Spufford, *The Great Reclothing of Rural England: Petty Chapmen and Their Wares in the Seventeenth Century* (London, 1984).

25 H. Newton, ‘Inside the sickchamber in early modern England: the experience of illness through six objects’, *English Historical Review*, cxxxvi (2021), 530–67; and J. Morgan, ‘Poverty and environment in early modern England’, in Hitchcock and McClure, *Routledge History of Poverty*, pp. 79–100.

26 T. LeCain, *The Matter of History: How Things Create the Past* (Cambridge, 2017).

27 S. Handley, ‘Lusty sack possets, nuptial affections and the material communities of early modern weddings’, *Environment and History*, xxviii (2022), 375–95, at p. 378.

28 Newton, ‘Inside the sickchamber’, p. 530.

29 L.M.A., COL/AD/01/011, *Letter Book L*, fols. 103v–104r.

30 Houston, *Featherbedds and Flocks Bedds*, pp. 83–4.

31 Horsehair was recognized to be a particularly itchy and uncomfortable bedding material, and could be deliberately used to disturb a person’s sleep (Richard Head, *The English Rogue* (4 vols., London, 1668–71), ii. 119).

32 Upholsterers Act 1495.

33 Handley, *Sleep*, p. 51.

34 Stuffing of Feather Beds, etc. Act 1551.

35 The valuation of feather beds based on weight is evident in probate inventories. See *Bristol Probate Inventories*, pt. i: *1542–1650*, ed. E. George and S. George (Bristol, 2002), p. 150.

36 L.M.A., Guild. Lib., MS30515, Transcription of upholders’ company charter, 14 June 1626, p. 1. The original charter was destroyed in the Fire of London in 1666. The charter was reissued in 1668 and the original version of this charter can be consulted at L.M.A., MS28374.

37 L.M.A., MS30515, p. 7.

38 L.M.A., MS30515, p. 8.

39 On the extension of company activities beyond the city of London, see J. Ward, *Metropolitan Communities: Trade Guilds, Identity and Change in Early Modern London* (Stanford, 1997), esp. pp. 27–44.

40 L.M.A., Guild. Lib., MS28375A, Transcription of bylaws of the Worshipful Company of Upholders, London, 1679, p. 16.

41 L.M.A., MS28375A, p. 7.

42 L.M.A., MS28375A, pp. 9–10.

43 Such flexibility was typical of livery company search procedures. See Berlin, ‘“Broken all in Pieces”‘, p. 82.

44 S. Pennell, ‘Consumption and consumerism in early modern England’, *Historical Journal*, xlii (1999), 549–64; and Weatherill, *Consumer Behaviour*.

45 Houston, *Featherbedds and Flocks Bedds*, p. 15.

46 L.M.A., MS07141/001, pp. 6, 294.

47 L.M.A., MS07141/001. Of the 462 mentions of particular fillings, ‘flocks and feathers’ appeared in 137 cases, ‘trash’ in 78, ‘squib’ in 55 and ‘coney wool’ in 47. These are only the instances in which these were the only materials present in a particular bedding item.

48 Upholsterers Act 1495; Stuffing of Feather Beds, etc. Act 1551; and L.M.A., MS30515.

49 Scalding is still used today in the poultry industry. See J. Kozák, ‘Goose production and goose products’, *World’s Poultry Science Journal*, lxxvii (2021), 403–14, esp. at p. 408.

50 H. Glasse, *The Art of Cookery made Plain and Easy* (2nd edn., London, 1747), p. 81.

51 N. Chomel, *Dictionnaire Oeconomique*, trans. R. Bradley (2 vols., Dublin, 1725), i. fol. Rrr r.

52 J. Kozák, I. Gara and T. Kawada, ‘Production and welfare aspects of goose down and feather harvesting’, *World’s Poultry Science Journal*, lxvi (2010), 767–78, esp. at p. 768.

53 N. A. Pluche, *Nature Delineated* (London, 1740), pp. 173–4.

54 Leonard Mascall, *The husbandlye ordring and gouernmente of poultrie* (London, 1581), fol. Fvi r.

55 E. Fudge, ‘Farmyard choreographies in early modern England’, in *Renaissance Posthumanism*, ed. J. Campana and S. Maisano (New York, 2016), pp. 145–66.

56 Mascall, *Poultrie*, fol. Fvi v.

57 Chomel, *Dictionnaire Oeconomique*, fol. Rrr r.

58 J. Macky, *A Journey Through Scotland in Familiar Letters From a Gentleman Here to His Friend Abroad* (London, 1729), pp. 29–30.

59 Lucas Jacobson Debes, *Faeroe, & Foeroa Referata* (London, 1676), p. 138.

60 Gideon Pierreville, *The Present State of Denmark* (London, 1683), p. 136.

61 Francis Willoughby, *The Ornithology of Francis Willoughby*, trans. J. Ray (London, 1678), p. 362.

62 Inventories typically indicate whether beds were stuffed with feathers or down (George and George, *Bristol Probate Inventories*, pt. i, p. 150).

63 R. Bradley, *A Philosophical Account of the Works of Nature* (London, 1721), p. 78.

64 Mascall, *Poultrie*, fol. Fvi v.

65 Tryon, *Treatise of Cleanness*, p. 5.

66 Handley, *Sleep*, p. 46.

67 Mascall, *Poultrie*, fol. Fvi v.

68 D. Woodward, ‘Straw, bracken and the Wicklow whale: the exploitation of natural resources in England since 1500’, *Past & Present*, clix (1998), 43–76, at p. 67.

69 *Seventeenth Century Economic Documents*, ed. J. Thirsk and J. P. Cooper (Oxford, 1972), p. 167.

70 Mascall, *Poultrie*, fol. Fvi v.

71 *The British Apollo, or, Curious Amusements for the Ingenious* (London, 1708), p. 7.

72 C. Hutton, *The Ladies Diary, or Woman’s Almanack, for the Year of Our Lord 1791* (London, 1791), pp. 25, 119.

73 Mascall, *Poultrie*, fol. Fvi v.

74 J. Morgan, ‘An emotional ecology of pigeons in early modern England and America’, *Environment and History*, xxviii (2022), 435–52, at p. 437.

75 Morgan, ‘Emotional ecology’, pp. 440–3.

76 L.M.A., MS07141/001, p. 156 (2). After the year 1703, the court minutes break and the volume details the company accounts. The page numbers restart for the 1704 courts. Where these pages are given, the number is succeeded with (2) to indicate the second set of page numbers.

77 L.M.A., MS07141/001, p. 19.

78 L.M.A., MS07141/001, pp. 168, 277.

79 ‘Cushion and bed terms’, in Randle Holme, *The Academy of Armory* (Chester, 1688), p. 97.

80 Tryon, *Treatise of Cleanness*, p. 11.

81 Tryon, *Treatise of Cleanness*, p. 5.

82 British Library, Lansdowne MS. 46/13, ‘Mr Peter Osborne, to Lord Burghley; to incorporate the Upholders, to prevent deceits in feathers, July 18, 1585’.

83 Tryon, *Treatise of Cleanness*, p. 5.

84 Tryon, *Treatise of Cleanness*, p. 8.

85 Robert Lovell, *Panzooryktologia* (Oxford, 1661), fol. C6 r.

86 Brit. Libr., Lansdowne MS. 77/41, ‘The Company of Upholsterers defend themselves against an accusation of their frauds in feathers, June 1594’.

87 Brit. Libr., Lansdowne MS. 77/42, ‘An answer of, the Upholsterers of London, to petition exhibited against them for their abuses in trade, 2 July 1594’.

88 W. Ellis, *The Modern Husbandman* (London, 1743), p. 81.

89 Like human hair, animal hair was understood as a form of excrement during this period, which may have contributed to its unsuitability for bedding (*A Culture History of Hair in the Renaissance*, ed. E. Snook (London, 2022), p. 97).

90 Stuffing of Feather Beds, etc. Act 1551; and L.M.A., MS28375A, p. 10.

91 T.N.A., MS. ADM 106/604/119, ‘Joshua Smith, London. Concerning the composition of bedding’, 18 Apr. 1705.

92 Handley, *Sleep*, p. 46.

93 L.M.A., MS07141/001, pp. 293, 4 (2).

94 L.M.A., MS07141/001, pp. 188 (2), 205 (2).

95 Edward Topsell, *The Historie of Foure-Footed Beastes* (London, 1607), p. 603.

96 Topsell, *Foure-Footed Beastes*, p. 625.

97 Topsell, *Foure-Footed Beastes*, p. 624.

98 Brit. Libr., Lansdowne MS. 77/41.

99 Brit. Libr., Lansdowne MS. 77/42.

100 Felix Platter, *Golden Practice of Physick* (London, 1664), p. 304.

101 Gervase Markham, *A Way to Get Wealth* (London, 1668), p. 107.

102 L.M.A., MS07141/001, p. 274.

103 L.M.A., MS07141/001, p. 22 (2).

104 L.M.A., MS07141/001, p. 63.

105 *Bristol Probate Inventories*, pt. iii: *1690–1804*, ed. E. George and S. George (Bristol, 2008), pp. 2–3.

106 George and George, *Bristol Probate Inventories*, pt. iii, p. 9.

107 *Documents Relating to the Colonial History of the State of New Jersey*, ed. W. Nelson (Trenton, N.J., 1901), p. 195.

108 Samuel Hartlib, *The Reformed Common-Wealth of Bees Presented in Severall Letters and Observations to Samuel Hartlib* (London, 1655), p. 17.

109 Trial of Ann Jaxon, 6 Sept. 1682, *Old Bailey Proceedings Online*, t16820906-7 <https://www.oldbaileyonline.org/record/t16820906-7?text=t16820906-7> [accessed 8 Feb. 2024]; and trial of Elizabeth Isham and Sarah Cullam, 4 Dec. 1717, *Old Bailey Proceedings Online*, t17171204-31 <https://www.oldbaileyonline.org/record/t17171204-31?text=t17171204-31> [accessed 8 Feb. 2024].

110 Trial of Richard Williams and Roger Griffis, 12 Oct. 1726, *Old Bailey Proceedings Onlne*, t17261012-16 <https://www.oldbaileyonline.org/record/t17261012-16?text=t17261012-16> [accessed 8 Feb. 2024].

111 L.M.A., MS07141/001, p. 74.

112 Company of Felt-Makers, *To the honourable the knights, citizens, and burgesses, in Parliament assembled* (London, 1691).

113 Worshipful Company of Skinners, *Reasons humbly offered* (London, 1691), p. 1.

114 M. Bailey, ‘The rabbit and the medieval East Anglian economy’, *Agricultural History Review*, xxxvi (1988), 1–20.

115 Bailey, ‘Rabbit’, p. 16.

116 J. Mortimer, *The Whole Art of Husbandry* (London, 1708), p. 189.

117 R. Sheail, ‘Rabbits and agriculture in post-medieval England’, *Journal of Historical Geography*, iv (1978), 343–55.

118 Bailey, ‘Rabbit’, pp. 6–7.

119 Bailey, ‘Rabbit’, p. 18; and K. Harvey, *The Imposteress Rabbit Breeder: Mary Toft and Eighteenth-Century England* (Oxford, 2020), pp. 19–20.

120 ‘APPENDIX to the CHRONICLE’, in *The Annual Register*, viii: *The Annual Register or a View of the History, Politicks, and Literature, of the Year 1765*, ed. E. Burke (London, 1765), pp. 189–90.

121 Robert Lovell, *Pambotanologia* (Oxford, 1659), p. 291.

122 Adam Olearius, *The Voyages and Travells of the Ambassadors* (London, 1669), p. 221.

123 H. Sloane, *A Voyage to the Islands of Madera, Barbados, Nieve, S. Christophers and Jamaica* (2 vols., London, 1707–25), ii. 73.

124 Lovell, *Pambotanologia*, p. 202.

125 W. Salmon, *Botanologia* (London, 1710), p. 463.

126 John Evelyn, *Sylva* (London, 1664), p. 47.

127 John Evelyn, *The Diary of John Evelyn*, ed. W. Bray (2 vols., London, 1901), i. 228.

128 Evelyn, *Sylva*, p. 58.

129 John Parkinson, *Theatrum Botanicvm* (London, 1640), p. 389.

130 Salmon, *Botanologia*, p. 463.

131 John Gerard, *The Herbal or Generall Historie of Plantes* (London, 1633), p. 1148.

132 Cambridge University Library (hereafter ‘C.U.L.’), GBR/0265/UA/VCCt.Invs 2, ‘Inventory of William Awgar alias Auger, Bedell, St Andrew’s Parish, 1557’.

133 Gerard, *Herbal*, p. 1148.

134 L.M.A., MS07141/001, pp. 137, 271.

135 Gerard, *Herbal*, pp. 45–6.

136 Salmon, *Botanologia*, p. 157.

137 Handley, *Sleep*, pp. 12–13; and L. C. Orlin, ‘Fictions of the early modern probate inventory’, in *The Culture of Capital: Properties, Cities and Knowledge in Early Modern England*, ed. H. S. Turner (London, 2002), pp. 51–83.

138 C.U.L., GBR/0265/UA/VCCt.Invs 6, ‘James Hartley Scholars Servant, 1601’.

139 C.U.L., GBR/0265/UA/VCCt.Invs 6, ‘Richard Meakes of Cambridge, barber surgeon lately deceased, 1604’.

140 Harley, ‘Consumption and material culture’, p. 204.

141 Brit. Libr., Lansdowne MS. 77/41.

142 John Pettus, *Fleta Minor* (London, 1686), p. 110.

143 Tryon, *Treatise of Cleanness*, p. 6. Tryon understood bedbugs to be generated by putrefaction in bedding (L. T. Sarasohn, ‘“That nauseous venomous insect”: bedbugs in early modern England’, *Eighteenth-Century Studies*, xlvi (2013), 513–30, at p. 516).

144 Brit. Libr., Lansdowne MS. 77/41.

145 C.U.L., GBR/0265/UA/VCCt.Invs 6.

146 Brit. Libr., Lansdowne MS. 77/42.

147 A. Logue, ‘“Saucy stink”: smells, sanitation and conflict in early modern London’, *Renaissance and Reformation*/*Renaissance et Réforme*, xliv (2021), 61–86.

148 John Monipennie, *The Abridgement or Summarie of the Scots Chronicles* (Edinburgh, 1671), pp. 257–8.

149 John Locke, *Some Thoughts Concerning Education* (London 1693), p. 23.

150 Morgan, ‘Poverty and environment’, p. 98.

151 Harley, ‘Consumption and poverty’, p. 90, Table 1.

152 E. Ash, *The Draining of the Fens: Projectors, Popular Politics, and State Building in Early Modern England* (Baltimore, 2017), p. 2.

153 Ash, *Draining of the Fens*, p. 28.

154 Ash, *Draining of the Fens*, p. 8.

155 *John Norden’s* The Surveyor’s Dialogue *(1618)*, ed. M. Netzloff (Farnham, 2013), p. 148. This was echoed in numerous other pro-drainage texts. See also P. Warde, ‘The idea of improvement, c. 1520–1700’, in *Custom, Improvement and the Landscape in Early Modern Britain*, ed. R. W. Hoyle (Farnham, 2011), pp. 127–48.

156 Ash, *Draining of the Fens*, p. 301.

